# Production of glycosylphosphatidylinositol-anchored proteins for vaccines and directed binding of immunoliposomes to specific cell types

**DOI:** 10.1590/1678-9199-JVATITD-2020-0032

**Published:** 2020-08-03

**Authors:** Wesley L. Fotoran, Nicole Kleiber, Thomas Müntefering, Eva Liebau, Gerhard Wunderlich

**Affiliations:** 1 Department of Parasitology, Institute of Biomedical Sciences, University of São Paulo, São Paulo, SP, Brazil.; 2 Max Planck Institute of Biophysics, Göttingen, Germany.; 3 Department of Molecular Physiology, Institute of Animal Physiology, University of Münster, Münster, Germany.

**Keywords:** Plasmodium, Liposome, Immune targeting, Vaccine

## Abstract

**Background:**

Liposomes are highly useful carriers for delivering drugs or antigens. The association of glycosylphosphatidylinositol (GPI)-anchored proteins to liposomes potentially enhances the immunogenic effect of vaccine antigens by increasing their surface concentration. Furthermore, the introduction of a universal immunoglobulin-binding domain can make liposomes targetable to virtually any desired receptor for which antibodies exist.

**Methods:**

We developed a system for the production of recombinant proteins with GPI anchors and histidine tags and Strep-tags for simplified purification from cells. This system was applied to i) the green fluorescent protein (GFP) as a reporter, ii) the promising *Plasmodium falciparum* vaccine antigen PfRH5 and iii) a doubled immunoglobulin Fc-binding domain termed ZZ from protein A of *Staphylococcus aureus*. As the GPI-attachment domain, the C-terminus of murine CD14 was used. After the recovery of these three recombinant proteins from Chinese hamster ovary (CHO) cells and association with liposomes, their vaccine potential and ability to target the CD4 receptor on lymphocytes in *ex vivo* conditions were tested.

**Results:**

Upon immunization in mice, the PfRH5-GPI-loaded liposomes generated antibody titers of 10^3^ to 10^4^, and showed a 45% inhibitory effect on *in vitro* growth at an IgG concentration of 600 µg/mL in *P. falciparum* cultures. Using GPI-anchored ZZ to couple anti-CD4 antibodies to liposomes, we created immunoliposomes with a binding efficiency of 75% to CD4^+^ cells in splenocytes and minimal off-target binding.

**Conclusions:**

Proteins are very effectively associated with liposomes via a GPI-anchor to form proteoliposome particles and these are useful for a variety of applications including vaccines and antibody-mediated targeting of liposomes. Importantly, the CHO-cell and GPI-tagged produced PfRH5 elicited invasion-blocking antibodies qualitatively comparable to other approaches.

## Background

Liposomal formulations are used for delivery of drugs or as vaccines [1, 2]. The success of liposome-based delivery depends mainly on the immense capacity of the used lipid composition to produce particles which then display specific features such as cationic external charge or PEGylated shields to produce stealth liposomes [3]. External layer modifications include immunoliposomes targeting specific cells using antibodies [4-6] and vaccinal liposomal particles [7]. Liposomal formulations may be employed to treat different diseases from cancer to bacterial and protozoal infections [8, 9]. However, several limitations in the formulation of liposomes include: i) chemical procedures with low efficiency, ii) directional orientation of antibodies, and iii) unspecific rescue of native antigens used in vaccine models [10-12]. 

To improve the production of cargo-loaded particles, we created an expression system that produces recombinant proteins easily associated with liposomes. The approach consists of a plasmid DNA vector that drives the production of recombinant proteins in eukaryotic cells. The desired protein is produced in frame with the C-terminus of murine CD14 containing an omega domain for GPI attachment, which results in an extractable membrane association of the recombinant protein. The purification is facilitated by histidine-tag (His-tag) or Strep-tag domains [13, 14] in association with physical enrichment of GPI-anchored proteins from the transfected cell line through the usage of Triton X114 [15]. The GPI moiety then promotes lipophilic association by co-incubation with liposomes. A comparable approach was used as an advanced form for a vaccine against different protozoan diseases but avoiding the association of multiple proteins without specificity [10,15-17,19]. Also, the association of a GPI anchor in co-incubation leads to the correct orientation on the outer layer of liposomes resulting in enhanced interactions with B cells for experimental vaccines [19]. Herein, we created three different proteins in fusion with the C-terminus of murine CD14 containing an omega domain that post-translationally receives a GPI anchor. These proteins were produced in CHO cells for different applications, as suggested before by other groups [16]. First, we produced a GFP-CD14 fusion (GFP-CD14-GPI_rec_) to monitor the technical viability and its association with liposomes. For the vaccine model, we created a CD14-fusion of the *Plasmodium falciparum* rhoptry protein 5 (PfRH5), which is probably the most promising vaccine candidate to date but reasonably difficult to obtain in recombinant form. Once purified as PfRH5-CD14-GPI_rec_, it was loaded on liposomes and immunized using MPLA (monophosphoryl lipid A) as an adjuvant [20]. Furthermore, we fused the ZZ domain [21], which is an artificial duplex of the *Staphylococcus aureus* IgG-Fc-binding domain, to CD14-GPI resulting in ZZ-CD14-GPI_rec_ to permit the tight association of antibodies to liposomes. Of note, the use of ZZ-CD14-GPI_rec_ allows the complexing of any commercially available antibody with an Fc domain. To demonstrate this principle, we tested the capacity of our immunoliposomes loaded with anti-CD4 antibodies to bind to CD4^+^ cells *ex vivo*.

## Methods

### ***Plasmodium falciparum* culture and growth inhibition assays**

*P. falciparum* parasites were cultured at 37°C in RPMI medium containing 0.5 % Albumax I (Life Technologies) in candle jars as described previously [22]. Synchronization of parasite blood stage forms was achieved by plasmagel flotation [23] followed by sorbitol lysis [24]. Growth inhibition assays were conducted in triplicate in 100 µL culture volumes at 3% hematocrit, starting with 1 % trophozoite stage parasites, supplementing the culture medium with 1:10 diluted sera from non-immunized mice or from proteoliposome-immunized mice. Parasitemias were counted by flow cytometry using ethidium-bromide-stained culture material after 24 h and 48 h growth, as described previously [25]. Growth inhibition was calculated by comparing parasitemias of cultures treated with purified antibodies from mice immunized against non-related proteins and those treated with antibodies from mice immunized with proteoliposomes containing PfRH5-CD14-GPI_rec_. In both cases, murine IgG antibodies from immunized mice were purified with protein G-agarose resin (Sigma), according to the manufacturer’s instructions.

### Construction of a vector for proteins with GPI fusion

The vector pcDNA3 (Invitrogen) was used in the construction of proteins with GPI anchors. First, the vector was modified to receive the TPA (tissue plasminogen activator) secretion signal. Next, the 6xHistidine-tag (His-tag) sequence and the NdeI/blunted-EcoR1 polylinker from pRSET A (Invitrogen) was inserted into the BamHI/blunted and EcoRI site. The final plasmid is called pcDNA3-A. All PCR-amplified fragments from each cloning step were initially A/T cloned in pGEM-T easy vector (Promega), according to the manufacturer’s instructions. Ligations were transformed and plasmids propagated in *Escherichia coli* DH10B cells. Sequences of amplicons cloned in pGEM were checked by semiautomatic Sanger sequencing. The omega motif from CD14 of *M. musculus* was amplified by PCR, targeting a fragment encoding the last 100 amino acids from the CD14 transcript (Primers used are listed in Additional file 1). 

The amplicon was cloned in the in the vector pcDNA3-A digested with EcoR1/NotI and ligated by using the same sites included in primers used to amplify the CD14 encoding region. For future purification of recombinant constructs, the Strep-tag was cloned after the 6xHis-tag by BglII/EcoRI restriction into the vector pcDNA3-A 6xHis, digested with BamHI/EcoRI. This DNA sequence was designed to include a novel BamHI site in 5’ of the EcoRI site in the fragment, containing a Strep-tag creating the plasmid pcDNA3-A-Strep-GPI, enabling the cloning of any fragment flanked by BamHI/EcoRI or compatible to these. Accordingly, PfRH5 and GFP-encoding sequences were amplified by primers containing a BamHI site in the 5’ region and an EcoRI in the 3’ region. PfRH5 was amplified using a plasmid template (donated by Dr. Alexander Douglas/Simon Draper, [26]) with codons optimized for eukaryotic expression and eGFP from a template, also codon optimized. The ZZ-domain was synthesized by GEONE-Brazil Technologies (sequence shown in Additional file 1). The GFP-coding sequence was cloned in a vector without Strep-tag, containing only the 6xHis-tag for purification. All PCR reactions consisted of 30 amplification cycles of denaturation at 94°C (1 minute), annealing at temperatures specific for each pair of primers (1 minute), polymerization at 72°C (1 minute) and a final polymerization at 72°C for 10 minutes. Taq Polymerase enzyme (Thermo/Fermentas) was used according to the manufacturer’s instructions.

### Preparation and solubilization of GPI-anchored protein from CHO cells

CHO cells were transfected (using JetPEI reagent, according to the manufacturer’s instructions) with modified pcDNA3-A for expression of GFP, PfRH5 or ZZ domains with the omega motif from CD14 for later GPI attachment and grown in RPMI with 5% fetal calf serum in 150 cm^2^ flasks under a 5% CO_2_ atmosphere. At 48 h after transfection, cells were harvested to obtain the insoluble membrane fraction. CHO pellets were obtained by centrifugation at 1200 x *g* for 15 min and maintained at −80 °C until required. Subsequently, the frozen CHO pellets were resuspended in TSCa buffer (50 mM Tris-HCl pH 7.25, 10 mM NaCl, 2 mM CaCl_2_), and sonicated at 4°C for 30 s at 240 W (Branson Sonifier). Then, a brief centrifugation (5 min) at 1000 x *g* was performed to sediment organelles, nuclei and intact cells. Subsequently, membrane fractions were isolated from the supernatant by ultracentrifugation at 100,000 x *g* for 1 h at 4 °C. The pellet was resuspended in TSCa buffer and ultracentrifuged again three more times under the same conditions described previously. Protein samples of the membrane fraction (diluted to 0.5 mg/mL) were solubilized in 1% Triton X-114 in TSCa for 1 h at 4 °C, under constant agitation, and briefly vortexed at 10-min intervals. After solubilization, the complex was submitted to centrifugation (30 min) at 2000 x *g* until it showed three distinct phases (insoluble pellet containing debris, GPI-enriched phase with Triton X-114, and supernatant). The middle, enriched phase was subsequently diluted in 10 volumes TSCa buffer, and used for conventional isolation of proteins with 6xHis and/or Strep-tag, according to the manufacturer’s instructions. The final protein solution was obtained in Imidazole Elution buffer (PBS with 500 mM NaCl and 0.5 M imidazole).

### Liposome and proteoliposome preparation

Liposomes and proteoliposomes were prepared with cholesterol and 1,2-dipalmitoyl-sn-glycero-3-phosphocholine (DPPC) in standard ratios of 1:4 [27]. The cholesterol and phospholipids in appropriate proportion (1 mg) were dissolved in 1 mL chloroform and dried under nitrogen flow. Afterwards, liposomes were loaded with fluorochrome DiA 4-(4-(dihexadecylamino)styryl)-N-methylpyridinium iodide (DiA; 4-Di-16-ASP) 20 µg/mg of DPPC lipid and dried together under nitrogen flow. The obtained lipid film was maintained under vacuum for 1 h. Then, 1 mL 50 mM Tris-HCl (pH 7.5) was added to the film. The mixture was incubated for 1 h at 60°C, above the critical temperature of the lipid, and briefly vortexed at 10-min intervals. Next, the lipid emulsion was sonicated for 2 min at 240 W, by means of a microtip (Branson Sonifier). The obtained mixture was centrifuged at 100,000 x *g* for 20 min at room temperature and the pellet was discarded. The supernatant containing small unilamellar vesicles was used to obtain proteoliposomes. For the immunization proposal, MPLA (from a 1 µg/mL stock suspension in water) in the total quantity of 25 ng per animal was added to the unilamellar vesicles in 50 mM Tris/HCl pH 7.5.

A protein sample obtained from the 6xHis-tag and/or Strep-tag purification phase (0.25 mg of total protein) was added to unilamellar vesicles with 1 mL of the previously obtained liposome fraction in 50 mM Tris/Cl pH7.5. The mixture was incubated overnight at room temperature. Finally, the resulting solution was centrifuged at 100,000 x *g* for 1 h, and the pellet preserved as proteoliposomes. For ZZ-domain proteoliposomes, the additional antibody was incubated together with ZZ-CD14-GPI_rec_, as were the control antibodies. Then, the mixture was submitted to ultracentrifugation. The pellet, constituted by proteoliposomes, was resuspended in 50 mM Tris-HCl (pH 7.5) and the supernatant was discarded. Importantly, the loading procedure of GPI-anchored proteins on preformed liposomes at room temperature likely results in an outer localization of GPI-anchored proteins, because the transition temperature of the herein used lipids is around 40°C (see datasheet of Polar Lipids).

### **Flow cytometer analyses of cells targeted *ex vivo* by DiA immunoliposomes**

Spleens from C57BL/6 mice were extracted from CO_2_-euthanized animals (Ethics Committee “CEUA” protocol number 015.3.3). After extraction of the spleen, total splenocytes were recovered and submitted to ACK buffer (154.4 mM ammonium chloride, 10 mM potassium bicarbonate, and 97.3 μM EDTA tetrasodium salt) for erythrocyte lysis. After three washing steps, the cells were stained with anti-CD4 Phycoerythrin (PE) and anti-CD8 PE for 30 min in PBS (pH 7.5) on ice. Only one antibody was used each time to avoid overlapping of fluorescence. Immunoliposomes containing membrane-staining DiA fluorochrome and anti-CD4 coupled by ZZ-CD14-GPI_rec_ were used for binding to total splenocytes for 15 minutes in PBS on ice. The control consists of liposomes with soluble ZZ-6xHis replacing the ZZ-CD14-GPI_rec_. After three washing steps, DiA fluorescence and PE of CD4 or CD8 cells were measured in a Guava EasyCyte HT flow cytometer (Merck Millipore, Darmstadt, Germany). The data were analyzed by the software Flow-Jo. Similar to this procedure, splenocytes were incubated with anti-CD4 coupled with DiA-stained ZZ-CD14-GPI_rec_ immunoliposomes, or the soluble ZZ version and stained with anti-CD4 allophycocyanin (APC) and anti-CD8-(APC) instead of PE-fluorochrome for immunofluorescence. The images of DiA- or APC-fluorescence from splenocytes were produced using a Zeiss Imager M2 fluorescence microscope.

### Flow cytometry analysis for the measurement of incorporation of GPI-anchored proteins and APC-marked antibodies bound to ZZ-CD14-GPI_rec_ to liposomes

To investigate the incorporation of GFP-CD14-GPI_rec_ on liposomes compared to GFP-GST_rec_, soluble proteins were produced by cloning the GFP gene in pGEX2T (Amersham Pharmacia) and subsequently GST-GFP_soluble_ was produced. After incubation with GFP-CD14-GPI_rec_ and GFP-GST_soluble_, proteoliposomes were centrifuged at 100,000 x *g*. Liposomes without proteins were measured in the first fluorescence channel (FL1 channel) and were set as zero fluorescence. Then, fluorescence of proteoliposomes with and without a GPI domain was analyzed. Measurement of fluorescence and incorporation of proteins was calculated by the following formula, resulting in the relative fluorescence index (RFI).

Fluorescence Index = number of proteoliposomes * geometric media from FL1 positive particlesµg of GFP protein used

The optimal loading of liposomes to form proteoliposomes was achieved by using 72.5 μg of ZZ-CD14-GPI_rec_ and 1 mg of lipids. For the formation of immunoproteoliposomes, the optimal conjugation of ZZ-CD14-GPIrec with anti-CD4 antibodies was determined by incubation with a FITC-labeled anti-mouse IgG. This was analyzed by flow cytometry after incubation with different quantities of FITC-labeled antibodies ranging from 1 μg to 1 ng to the ZZ domain. The maximal occupation of antibodies to the ZZ domain was determined by increasing the IgG amount until the geometric fluorescence reached a plateau.

### Dynamic light scattering analysis of proteoliposomes

The liposome and/or proteoliposome size distribution was determined by dynamic light scattering using a Malvern Zetasizer µV apparatus (Malvern Panalytical, UK) following the manufacturer's instructions. The sample was filtered and diluted, and its polydispersion index was measured.

### ELISA and IgG isotypes

Ninety-six well ELISA plates were coated with 250 ng/well merozoite extract from *P. falciparum* at 4°C overnight in 50 mM sodium carbonate buffer (pH 9.8). On the next day, plates were washed with PBS/0.1 % Tween 20 and blocked with 4 % nonfat milk/PBS for 2 h at RT. Equal quantities of primary antibodies from pre-immune and immunized mice were used starting with a 1:300 dilution, which was serially increased in order to determine the detection limit in an endpoint ELISA format. Antibodies were incubated for 2 h at RT. After four washing steps, a secondary anti-mouse IgG-HRP antibody (1:5000) was used for the subsequent colorimetric reaction. After washing, all wells were developed with TMB substrate (Pierce) and stopped with 1 M HCl; the result of the colorimetric reaction was analyzed in a BioTek plate reader at 450nm/595nm. The cutoff value was determined as the average signal obtained with pre-immune sera plus two standard deviations.

### Cryoelectron microscopy

Cryoelectron microscopy was performed as described previously [28]. Briefly, nanoparticles were analyzed by spotting 3 µL of the specimen to a holey carbon-film grid (Quantifoil Micro Tools GmbH, Jena, Germany) pretreated with Gatan Solarus 950 plasma cleaner. The grid was shock-frozen in liquid ethane using a Gatan Cryoplunge 3 (Gatan Inc, Pleasanton, CA, USA). Low dose imaging of the frozen, hydrated specimen kept at liquid nitrogen temperature with a Gatan 626 single tilt cryoholder was performed in a JEM2100 electron microscope (JEOL Ltd, Tokyo, Japan) operating at 200 kV. All images were recorded at ~40,000 x magnification using a Gatan UltraScan 4000 CCD camera.

### Immunization schedules

Male BALB/c mice and C57BL/6 mice, 8-12 weeks old, were bred and maintained under standard conditions in the animal facility at the Institute for Biomedical Sciences, University of São Paulo. Mice were treated in accordance with national animal welfare regulations and the experimental protocol was approved before the actual experiments as stated above. Five animals per formulation were immunized intraperitoneally (i.p.) on days 0, 14, 28, using 10 μg of protein in 100 μl Tris/HCl buffer for each immunization with 25 ng of MPLA/animal. Serum samples were taken on days 0 (pre-immune sera), 14, 28 and 42 days. For antibody titer determination, the samples from all four blood extractions were tested by standard ELISA for reactivity with the recombinant antigen PfRH5-GST produced as previously described [29]. Sera with positive reaction were used for subsequent IgG purification. Recombinant antigens were produced as previously described [29].

### Immunofluorescence

Live cell immunofluorescence assays were conducted with CHO cells expressing GFP after transfection and selection by the antibiotic G418 (600 µg/mL) [30]. When necessary, proteins were detected by specific monoclonal antibodies against 6xHis-tag (Abcam, Anti-6x His tag® antibody [HIS.H8] (ab18184)). For generation of antiPfRH5 antibodies, mice were immunized with MPLA-adjuvanted proteoliposomes, loaded with PfRH5-CD14-GPI_rec_ proteins. In order to test PfRH5 antigen presence in parasites or in CHO cells, trophozoite/schizont stage parasites or CHO cells were incubated with 40 μg/mL of 4’-6-diamidino-2-phenylindole (DAPI) in 100 μL RPMI/1% bovine serum albumin for 60 min at 37 °C, followed by primary antibody at a dilution of 1:250 and an Alexa Fluor 594 labeled anti-mouse-IgG or FITC (Molecular Probes, 1:500 dilution). Between each incubation step, the material was washed three times with RPMI adjusted to pH 7.2 with 0.23% NaHCO_3_. In the case of parasite detection by anti-PfRH5 antisera, pre-immune sera from the group of mice which afterwards received proteoliposome immunization, were used as a negative control. Immunofluorescence was visualized on a Zeiss Imager M2 fluorescence microscope. For ER/Golgi visualization, the dye ER-Tracker™ Blue-White DPX (20 µg/mL, for live-cell imaging, Invitrogen) was used (emission 488 nm). Due to its incompatibility with DAPI (same spectral emission wavelengths) ethidium bromide in 2 µg/mL (emission 640 nm) was added for nuclei visualization and analyzed by the same excitation of ER/Golgi stain (488 nm).

## Results

### CHO cells transfected with pcDNA3-A-GPI produce partially surface-localized proteins with intact purification tags

**Figure 1.**
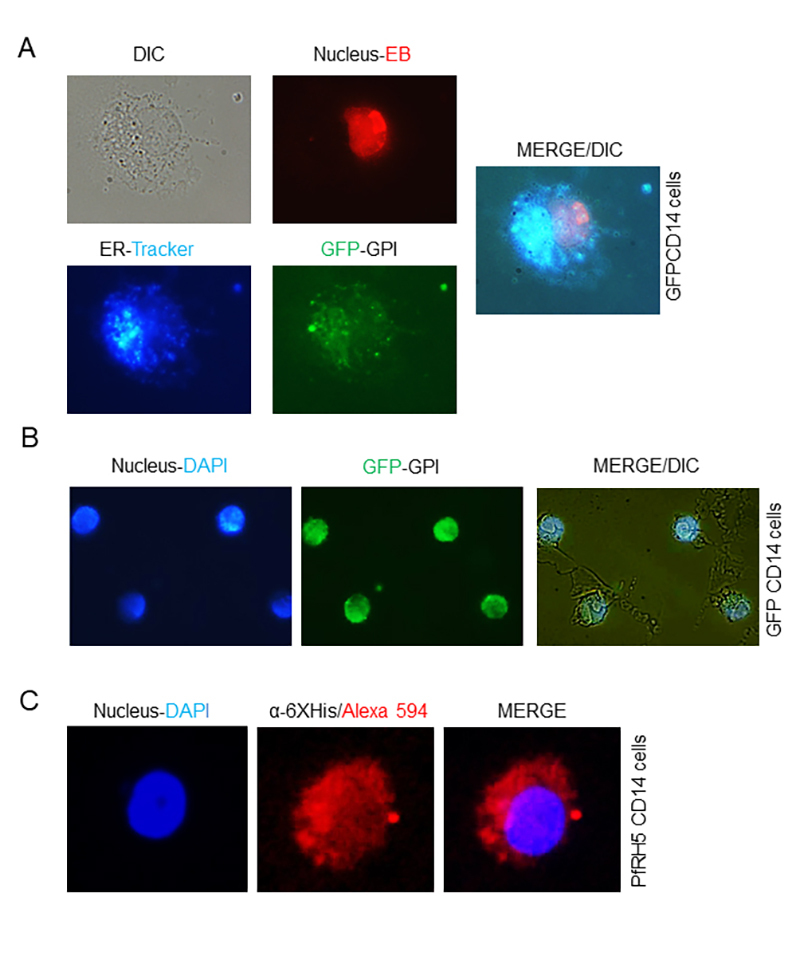
CHO-cells express GFP-CD14 or PfRH5-CD14 inside cells and on their surface after transfection. **(A)** Representative cells expressing different recombinant proteins with GPI anchor in the endoplasmic reticulum/Golgi (ER) and partially on the cell surface. CHO cells transfected with pcDNA3-GFP-CD14-GPI were stained with ethidium bromide (EB, stains DNA) and ER-Tracker. For localization, the GFP fluorescence was observed at the same excitation for all markers, showing areas of colocalization between GFP and ER-tracker. DIC indicates the clear field image. **(B)** The same CHO GFP-CD14-expressing cells were stained with DAPI for visualizing nuclei, and GFP fluorescence is visible throughout the cell and on the cell surface. **(C)** Cells transfected with PfRH5-CD14 with 6xHis/Strep tags were stained with DAPI, whereas the presence of PfRH5-CD14 around the cells was highlighted by incubation with ant-6xHis as primary antibody and a secondary antibody anti-mouse IgG conjugated to Alexa 594. Wild type CHO cells showed no significant fluorescence when exposed to anti-6xHis and the anti-mouse Alexa 594 (data not shown).

To verify whether the pcDNA3A constructs lead to the production of cell-surface localized proteins linked to GPI anchors, we transfected CHO cells with pcDNA3-A GFP-CD14 and pcDNA3-A PfRH5-CD14 plasmids. These were then analyzed for GFP fluorescence to evaluate the localization in cellular compartments. The cells expressing GFP-CD14-GPI_rec_ showed green fluorescence in the same location as the ER/Golgi apparatus evidenced by colocalization of ER/Golgi stain, which indicates secretion of GFP-CD14-GPI_rec_ (Figure 1A). To verify whether the protein also appears on the cell surface, we washed the cells three times with PBS and stained the cells with DAPI. The cells showed GFP-fluorescence on or near their surface (Figure 1B). We also examined surface expression of PfRH5-CD14-GPI_rec_. For this, transfected cells expressing the 6xHis-tagged PfRH5-GPI_rec_ were stained with anti-6xHis antibodies. The results show that PfRH5-CD14-GPI_rec_ is located on the surface of CHO-cells and remains there as an intact polypeptide for purification via Strep- and His-tags (Figure 1C).

### Liposome loading with GFP-CD14-GPI_rec_ is more efficient than with GFP-GST

**Figure 2.**
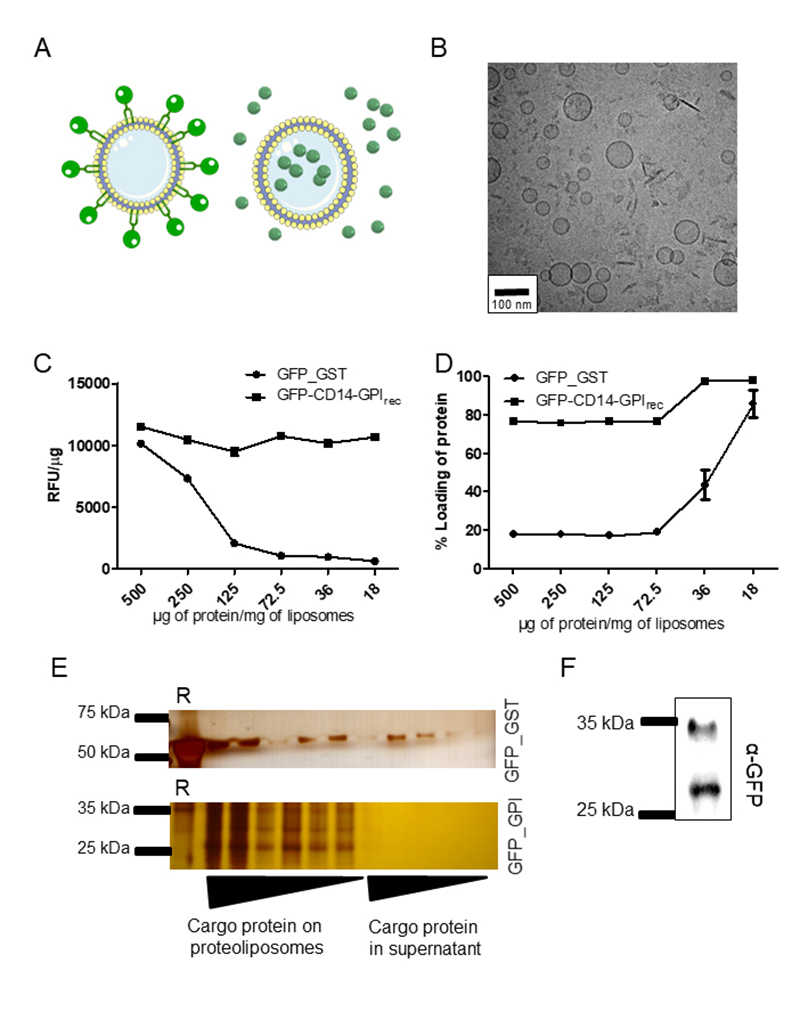
Incorporation of GFP-CD14 onto liposomes by its lipophilic domain is superior to incorporation of GFP in liposomes by simple co-incubation. **(A)** Proposed model of liposomes with either GFP fused to CD14-GPI_rec_ localizing outside of liposomes (left side) or soluble GST-GFP (right side). **(B)** Cryo-TEM fracture images of proteoliposomes formed after incubation with GFP-CD14-GPI_rec_ (72.5 µg of protein/mg lipid). **(C)** To show dose-dependent incorporation of recombinant proteins, the relative fluorescence in proteoliposomes emitted by GFP (Relative fluorescence units, RFU) was analyzed by flow cytometry and normalized to the amount of protein used to form proteoliposomes (RFU/µg of protein). The amount of protein used to form proteoliposomes was varied (ranging from 500 µg to 18 µg) while maintaining a fixed quantity of 1 mg of total lipids to form liposomes. This experiment was done twice in triplicate. **(D)** Formed proteoliposomes were ultra-centrifuged and the amount of protein incorporated in each batch was measured in proteoliposomes (pellet) and in the supernatant fraction. The amount of protein retained in proteoliposomes from incubation of GFP-GST (soluble form of GFP) or GFP-CD14-GPI_rec_ (membrane-attached form of GFP extracted from transfected CHO cells) is expressed as a percentage of protein loaded in proteoliposomes. This experiment was also done twice in triplicate. **(E)** To demonstrate a dose-dependent incorporation of recombinant proteins/proteoliposomes obtained after ultracentrifugation, aliquots from pellets of proteoliposomes and supernatants were submitted to SDS-PAGE electrophoresis followed by silver staining. The complete pellet fraction of formed proteoliposomes and 20 µL of the supernatant of each batch were applied in a sequence. The first lane of each gel is the recombinant protein used in each experiment alone (“R”). **(F)** GFP on proteoliposomes can be detected by anti-GFP antibodies. The proteoliposome used was obtained from a batch loaded with 72.5 µg GFP-CD14-GPI_rec_ per mg lipid after ultracentrifugation. Note that GFP is partly uncoupled from the CD14-GPI peptide leading to full-length (upper band) and GFP-only protein species (lower band). Empty liposomes receiving the same treatment showed no signal with the anti-GFP used herein (data not shown).

The association of GPI-anchored proteins may alter the physicochemical structures of the liposome surface leading to a different average size and size range compared to empty or GFP-GST loaded liposomes (proposed model Figure 2A). Accordingly, we observed that GFP-CD14-GPI_rec_ or PfRH5-CD14-GPI_rec_, formed liposomes augmented in size and dispersity. As demonstrated in Table 1, the average diameter size of loaded particles was approximately 170 nm with a size range from 140-240 nm, while unloaded liposomes presented an almost uniform size around 70 nm. In contrast, cryoelectron microscopy images of the final particles, loaded with GPI-anchored proteins showed a particle size of under 100 nm (Figure 2B). This shows that the light scattering is an appropriate marker for protein incorporation but does measure the correct size of the liposome core. To analyze whether the GPI anchor is sufficient to attach proteins to liposomes in a simple co-incubation, we analyzed the increase in fluorescence of each liposome. This was done for the GFP-CD14-GPI_rec_ protein (termed here GFP--GPI_rec_) and a soluble form of GFP fused to GST. The particles exposed to GFP-GPI_rec_ showed a higher fluorescence signal compared to those exposed to GFP-GST per µg of protein, as shown in Figure 2C.

**Table 1.** Size and dispersity index of liposomes loaded with GFP-CD14-GPI_rec_. Values for GFP-CD14-GPI_rec_ and empty liposomes are shown.

Liposomal particle	Size	PDI (polydispersity index)
**Liposomes**	70 (+/-20) nm	0.2 (+/- 0.02)
**Proteoliposomes**	170 (+/-70) nm	0.5 (+/- 0.1)

The same experiment was performed to analyze the quantity of protein incorporated by proteoliposomes. The amount of protein loaded into proteoliposomes is higher for GFP-CD14-GPI_rec_ than for soluble GFP-GST (Figure 2D). The corresponding western blot confirmed this result (Fig. 2E). To demonstrate the efficiency of incorporation, vesicles were analyzed in immunoblots using antibodies against GFP. As a result, proteoliposomes showed incorporation of GFP-CD14-GPI_rec_, which resisted three washing steps (Figure 2F). This indicates that the construct can be utilized to purify specific proteins and load them on liposomes. Furthermore, after all purification steps, the GFP-CD14-GPI_rec_ retains fluorescent properties. Taken together, these assays allowed determination of the optimal relation of 75 µg protein per mg of lipid for co-incubation with CD14-fused proteins, probably containing a GPI anchor.

### Proteoliposomes containing PfRH5-CD14-GPI_rec_ induce a robust humoral response and resultant IgGs exert growth inhibition activity

**Figure 3.**
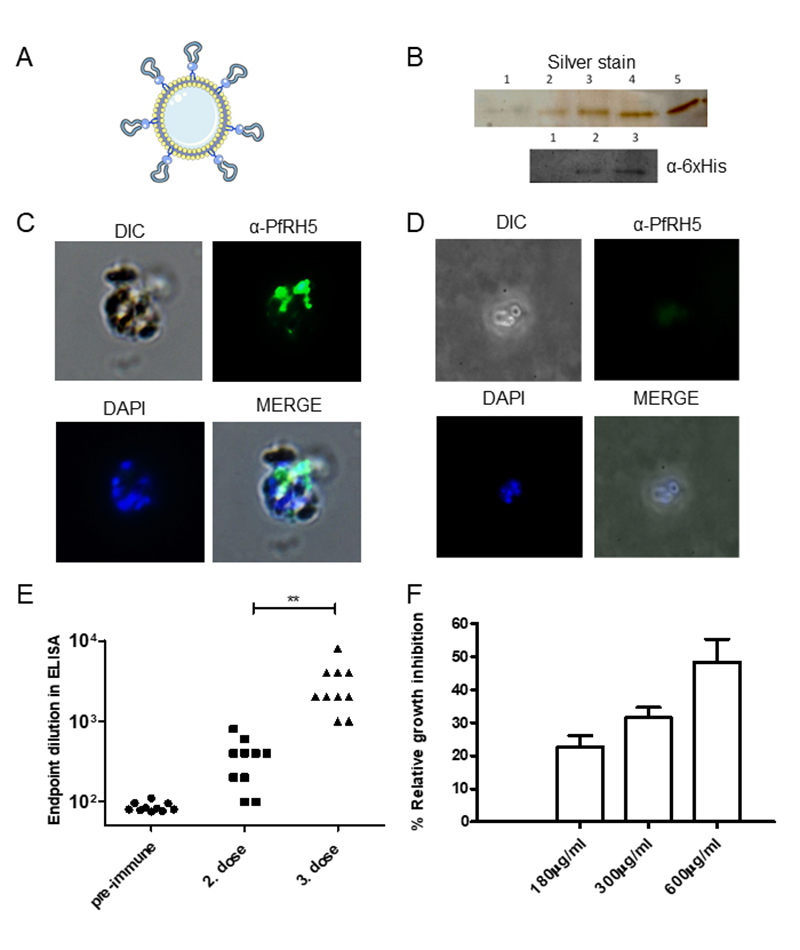
Formation and characterization of proteoliposomes with PfRH5-CD14-GPI_rec_ for vaccine use against *P. falciparum*. **(A)** Proposed model of PfRH5-CD14-GPI_rec_-loaded liposomes. **(B)** Proteoliposomes loaded with the antigen PfRH5-CD14-GPI_rec_ were produced and submitted to SDS-PAGE and proteins were visualized by silver stain (upper gel). In Lane 1, the supernatant of the proteoliposome production was applied. Lane 2 is a sample of PfRH5-CD14-GPI_rec_ used in proteoliposome production. Lanes 3, 4 and 5 represent aliquots of three different proteoliposomes used in immunizations. In the lower gel, an antibody against the 6xHis tail was employed to detect the recombinant form of PfRH5-GPI in proteoliposomes (Western blot). Lane 1 shows empty liposomes, Lane 2 the recombinant form of PfRH5-CD14-GPI used to produce proteolipossomes, and Lane 3 material from proteoliposomes utilized to immunize mice. Visualized or detected bands show the expected molecular weight around 50 kDa. **(C)** *P. falciparum* schizonts were analyzed by immunofluorescence to verify whether IgG in the sera from immunized mice recognized the native form of PfRH5 in rhoptry structures from fresh parasites. **(D)** The same immunofluorescence approach was applied to pre-immune sera to reveal the absence of unspecific recognition. **(E)** Sera from animals (n = 10) were used in ELISA assays against late schizont extracts of *P. falciparum* and the pre-immune sera were used as to establish a cutoff for reactivity in ELISA after the second and the third dose of immunization. A third dose increased antibody responses in immunized mice (paired *t* test, ** is p < 0.01). **(F)** Sera from immunized mice were used in *in vitro* assays to verify whether the antibodies generated after immunization were able to inhibit reinvasion of *P. falciparum.* Different quantities of individually purified IgG showed a dose-dependent inhibitory effect at 180 µg/mL, 300 µg/mL and 600 µg/mL IgG, with matched quantities of pre-immune IgG. Shown are the average values from 10 individual IgG preparations.

In the following assay, we used purified PfRH5-CD14-GPI_rec_ from CHO cells, incubated with liposomes, as antigens for immunization (for a model, Figure 3A). Supposedly, the CD14-omega domain with its GPI anchor from *M. musculus* is not immunogenic in mice. In order to enhance the immune response, MPLA was used with PfRH5-CD14-GPI_rec_ proteoliposomes. We observed that PfRH5-CD14-GPI_rec_ protein was abundantly detectable on proteoliposomes after incubation, indicating that the conjugation to the CD14-domain with GPI moiety can also attach larger proteins such as PfRH5 to liposomes (Figure 3B). After immunization, IgGs present in the immune sera were able to recognize live forms, especially merozoites, while pre-immune sera were not (Figure 3C and 3D). IgGs from immunized animals recognized targets in a *P. falciparum* merozoite extract, as shown by ELISA. All animals were able to respond to native antigens with an average endpoint titer of 10^3^-10^4^ (Figure 3E). Finally, we tested the capacity of IgGs containing anti-PfRH5 to inhibit parasite growth *in vitro.* Using a dose-response assay (range from 180-600 µg/mL IgG), we observed that parasites were growth-inhibited by addition of IgG, compared to pre-immune sera. The maximum inhibition was 45-60% at 600 µg/mL (Figure 3F). These results reveal that the vaccine candidate PfRH5 can be attached to liposomes via the GPI of murine CD14 and that this conjugate - displayed on liposomes - is highly effective in creating functional antibodies against PfRH5.

### Immunoliposomes with ZZ-CD14-GPI_rec_ bind on specific cell targets and show less unspecific binding on non-target cells

**Figure 4.**
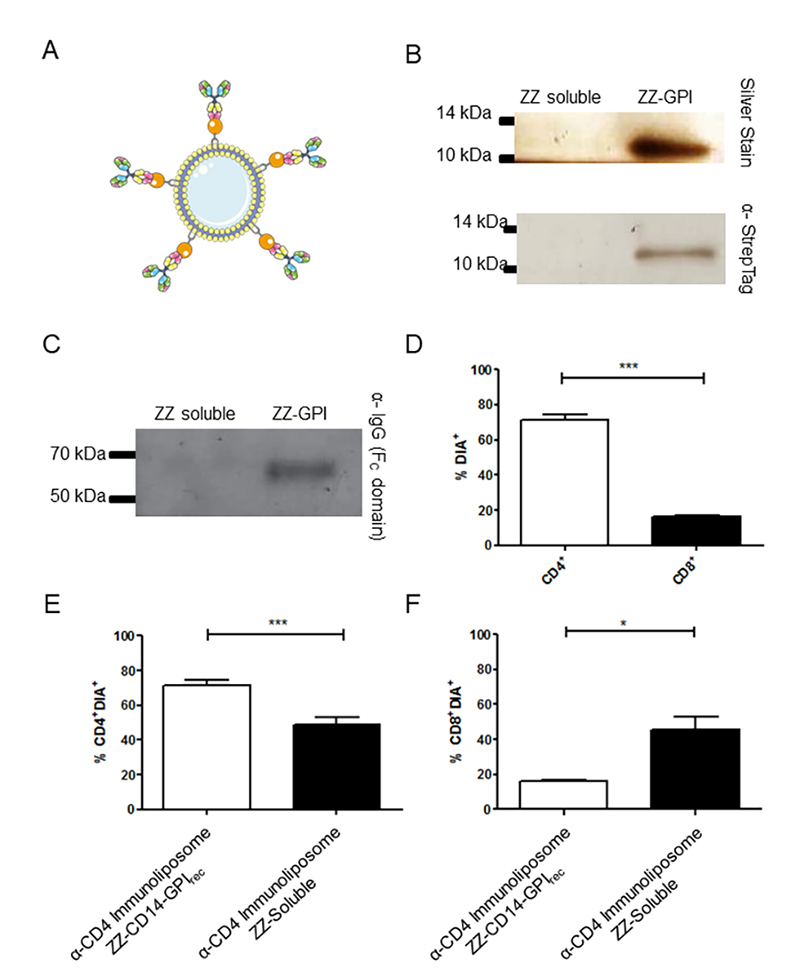
ZZ-CD14-GPI_rec_ function to associate anti-CD4 with liposomes. **(A)** Proposed model of liposome-resident ZZ-GPI_rec_ binding to antibodies. **(B)** The incorporation of ZZ-GPI_rec_ on proteoliposomes was analyzed by silver-stained SDS-polyacrylamide gels and western blot in pelleted proteoliposomes produced by co-incubation with ZZ-CD14-GPI_rec_, or soluble ZZ as a control using the indicated antibodies. **(C)** To analyze whether a second incubation of proteoliposomes with antibodies is viable for linking antibodies to proteoliposomes, pellets of proteoliposomes produced with ZZ-GPI were placed with ZZ in a soluble version after incubation with anti-CD4 antibodies. The proteoliposomes of this incubation were submitted to a western blot using an antibody against the F_C_ domain of IgG. **(D)** Liposomes loaded with ZZ-CD14-GPI_rec_ and anti-CD4 were incubated with DiA, washed and exposed to splenocytes from BALB/c mice (n=5). The CD4^+^ and CD8^+^ splenocytes were differentiable, each being detected separately with anti-CD4 or anti-CD8 antibodies coupled to APC (APC fluorochrome emission at 660nm). Total splenocytes were analyzed by flow cytometry for each individual marker as CD4^+^ and CD8^+^ cells. The quantity of double-stained CD4^+^ cells with DiA^+^ and CD8^+^ cells with DiA^+^ were analyzed by a paired *t* test. A significantly higher number of immunoliposomes interacting with CD4^+^ cells compared to CD8^+^ cells was observed (*** is P<0.005). In **(E)** the capacity to mediate binding to CD4^+^ of ZZ-CD14-GPI_rec_-loaded liposomes associated with anti-CD4 was verified. The soluble ZZ version associated with anti-CD4 was used as a control for passive transference/fusion of DiA-stained liposomes to cells. The amount of CD4/DiA-stained cells was significantly higher when the immunoliposomes contained the CD14 (and thus probably the GPI anchor) fused to the ZZ domain (paired *t* test, *** is p < 0.005). **(F)** The potential off-target labeling was checked by flow cytometry monitoring colocalization of ZZ-GPI_rec_ or ZZ pre-incubated with anti-CD4 plus DiA with APC-anti-CD8 labelled splenocytes. In this case, soluble ZZ-anti-CD4/DiA liposomes colocalized more with CD8^+^ cells than the GPI-anchored ZZ-antiCD4 complexes (paired *t* test, * is p < 0.05).

In the following procedure, we used the IgG domain of protein A from *S. aureus* fused in two copies (ZZ) to the CD14 omega domain to produce a ZZ-CD14-GPI_rec_ protein, which is able to bind antibodies to liposomes (for a model see Figure 4A). The ZZ-CD14-GPI_rec_ protein could be detected on proteoliposomes as predicted, compared to the soluble version of the ZZ domain, which did not bind to liposomes (Figure 4B). Next, ZZ-CD14-GPI_rec_ loaded liposomes were incubated with anti-CD4 antibodies. Only proteoliposomes with ZZ-CD14-GPI_rec_ showed association of antibodies as seen in western blots, ruling out any spurious interaction of antibodies with empty liposomes (Figure 4C). Then, we determined whether ZZ-CD14-GPI_rec_ proteoliposomes loaded with anti-CD4 antibodies were able to bind to CD4^+^ cells. For this, splenocytes obtained from C57Bl/6 mice were incubated with anti-CD4-loaded ZZ-CD14-GPI_rec_ proteoliposomes that were preincubated with the fluorescent membrane marker DiA. After incubation, bulk splenocytes were stained with anti-CD8-PE and anti-CD4-PE; the CD8^+^ cells function here as a control for unspecific binding and should therefore show less double staining with DiA and PE. Accordingly, the use of ZZ-CD14-GPI_rec_ increased the amount of doubly stained DiA/PE CD4^+^ cells when compared to CD8^+^ cells, which showed less DiA/PE staining (Figure 4D). It is possible that DiA passively diffuses from DiA-labeled liposomes to lymphocytes, leading to positive double staining with DiA/PE. To investigate this, we performed the same assay with a soluble version of ZZ without the CD14-omega domain. An increase of DiA fluorescence was detectable in CD4^+^ cells with ZZ-CD14-GPI_rec_ loaded and anti-CD4-associated liposomes when compared to the soluble ZZ version (Figure 4E), which also showed significant staining of cells. Of note, ZZ-CD14-GPI_rec_ incorporation protected the liposomes from unspecific transfer of the DiA signal to splenocytes in *ex vivo* conditions. This was tested using the same construction with ZZ-CD14-GPI_rec_ or a soluble ZZ version where more CD8-positive cells became DiA-stained when soluble anti-CD4-ZZ complexes were employed (Figure 4F), indicating that the anti-CD4-ZZ-CD14-GPI_rec_ complex shields the membrane-associated DiA from diffusing to CD8^+^-cells or decreases the fusion of empty, DiA loaded liposomes to CD8^+^ cells.

**Figure 5.**
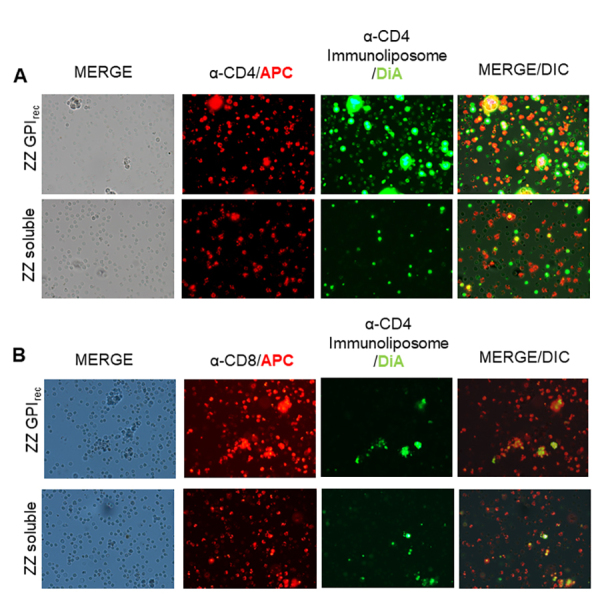
Visualization of immunoliposome-binding to total splenocytes. **(A)** Immunofluorescence of splenocytes stained with a CD4^+^ marker after incubation with immunoliposomes produced with ZZ-GPI_rec_ and ZZ in a soluble version. CD4+ cells were stained in red, while cells stained by α-CD4 immunoliposome are shown in green; colocalization of both markers is indicated in yellow. Colocalization mainly occurs with ZZ-GPI bound anti-CD4 compared to soluble ZZ. **(B)** To verify the unspecific DiA staining by α-CD4 immunoliposomes, CD8+ cells were stained with APC. As above, ZZ-GPI-antiCD4 loaded liposomes and soluble ZZ-antiCD4 mixed with liposomes were exposed to cells and no colocalization difference was detected, demonstrating the colocalization background in this test.

To further establish the interaction of anti-CD4-ZZ-GPI_rec_-DiA-loaded liposomes with CD4+ splenocytes, we tested the colocalization of these with anti-CD4-APC stained splenocytes. As expected, anti-CD4-APC partially stained the splenocytes and only the anti-CD4-ZZ-GPI_rec_ liposomes labeled with DiA were able to stain the same number of cells stained with anti-CD4-APC. In the overlay image, both markers DiA and α-CD4-APC appeared in partial colocalization. However, in parallel DiA stained liposomes plus soluble anti-CD4-ZZ did not show the same degree of colocalization (Figure 5A). This underscores the importance of the GPI-domain to anchor the antibodies to liposomes and enable their interaction with the CD4^+^ cell target. To confirm whether this was specific for CD4^+^-cells and no other targets, anti-CD8^+^ APC-stained antibodies were utilized to stain splenocytes and these were incubated with anti-CD4-ZZ-CD14-GPI_rec_/DiA-stained immunoliposomes. As shown in Figure 5B, anti-CD8^+^-APC-positive cells were rarely stained with DiA-stained anti-CD4-ZZ-GPI_rec_ liposomes or with soluble versions of the anti-CD4-ZZ protein complex. This reinforces that apparent off-targeting to CD8^+^ cells by anti-CD4-ZZ-CD14-GPI_rec_ liposomes is due to passive DiA transference and not an effect mediated by antibodies which are present on ZZ-CD14-GPI_rec_ liposomes.

## Discussion

Bioengineering of recombinant proteins has contributed to the development of important applications in many fields [31-35]. In parallel, the engineering of biocompatible nanoparticles, such as liposomes, opened different avenues for multiple treatments ranging from pharmacological applications [36] and diagnostic techniques [27] to vaccine design [37-39]. In order to combine both techniques, the conjugation of protein to modified lipids represents a promising new strategy [40, 41]. However, problems that arise when loading recombinant proteins in liposomes include low loading efficiency, time-consuming protein manufacturing and incorrect orientation of the associated cargo proteins [42, 43].

An alternative that can resolve some of these problems is producing proteins with lipophilic domains. Several studies already reported promising results in creating techniques to improve the correct insertion of proteins in external layers of liposomes [44-47]. For immunological applications, the correct interaction of proteins and lipids plays a critical role in order to warrant efficient immunization [48]. The GPI anchor is a widely used strategy in nature to fix proteins on lipid layers [46]. In several cases, these proteins play important roles for protozoan pathogens such as *Plasmodium* [10, 15, 49], *Leishmania* [27] and *Trypanosoma* [50], and these proteins may directly be exploited for vaccine use. For example, in the *Plasmodium* model, high titers of antibodies were generated by the association of liposomes and native GPI antigens, creating directed proteoliposomes with antigens outside of these particles, apparently able to interact with and stimulate B cells [15, 18, 49]. The setup used in these studies had the disadvantage of including several unknown GPI-anchored antigens but not specific ones that are on the merozoite surface from *Plasmodium* [10, 15]. In total, 30 GPI-anchored proteins were predicted to exist in *P. falciparum*, 11 of which were detected in schizonts/merozoites [51]. For the sake of specificity, we created and tested a system that is able to produce one desired specific protein linked to the murine CD14-omega domain that then leads to a surface localization of the desired protein, which is then extracted by Triton X114, purified by affinity tags such as strep- or His-tags and later placed on liposomes by simple co-incubation. Although we did not attempt to detect the GPI-domain on our constructs, we assume that it is present. Otherwise, the herein produced proteins would have been secreted into the cell supernatant and not remained in association with cells. Furthermore, the results of experiments shown in Figure 4 highlight that CD14-containing and therefore probably GPI-containing proteins associated with liposomes while similar proteins not containing the omega domain of CD14 were not detected. 

After testing optimal extraction methods, it was important to prove that the extracted and liposome-transferred proteins maintained at least a partially correct conformation, which is mandatory for vaccine applications. For this, the antigen PfRH5 was used. PfRH5 is a very promising target for an inhibitory humoral response in *P. falciparum* [27] and different studies used either recombinantly expressed or adenovirus-produced PfRH5. Our approach was able to elicit antibodies that recognized native and recombinant forms of PfRH5 and also observe inhibition of parasite growth/reinvasion. The observed inhibition was comparable to other trials [27]. Of note, PfRH5 is of pivotal importance for merozoite invasion by its interaction with Basagin-containing erythrocytes [49, 52, 53]. 

The delivery of molecules to defined cell types or tissues is an elegant way to enhance the therapeutic effect of drugs or other effector molecules. In order to enable the directing of liposomes to specific receptors, the omega domain of CD14 was fused to the Fc-binding ZZ polypeptide, resulting in a membrane-associated form of ZZ. Using CD4^+^ cells as a target, we observed that about 70% of CD4^+^ cells could be targeted by ZZ-CD14-GPI_rec_ liposomes associated with anti-CD4 under *ex vivo* conditions. We chose CD4^+^ cells due to their key roles in the immune system, being for example the reservoir of HIV but also an interesting target for immune therapies approaching exhaustion of CD4^+^ cells due to malaria [11, 54-56]. Of note, CD8^+^ cells and CD4^+^ cells are similar in form, size and location. Anti-CD4 directed liposomes had a lower unspecific binding to CD8^+^ cells, showing accuracy in targeting CD4^+^ cells. The selective targeting toward CD4^+^ cells and few off-targets on CD8^+^ cells prove that immunoliposomes employing a ZZ-CD14-GPI anchor are useful for specific targeting of cells, considering that virtually any antibody can be coupled to form immunoliposomes by anchoring on the external layer of liposomes.

## Conclusions

The fusion of the CD14 omega domain with the vaccine-relevant malarial antigen PfRH5 and its delivery in the form of liposomes provided an efficient humoral immune response against the *P. falciparum* parasites as evidenced by growth inhibition assays. We envision that the fusion of other proteins or peptides, such as the receptor-binding domain of the new Coronavirus CoV-2019 spike protein, and their association with liposomes may also be feasible and efficient as an immunogen. In addition, the fusion of a universal IgG Fc-binding domain via the same CD14 domain is viable. The insertion of this fused construct into liposomes permits the association of specific IgGs targeting possibly any structure. Probably, the loading of drugs into these directable liposomes may enhance their therapeutic effect against important diseases such as cancer while decreasing unwanted side effects.

## Supplementary material

Additional file 1. Sequences of the synthesized ZZ-domain and of oligonucleotides used for murine CD14 amplification (introduced restriction sites are underlined).

## References

[B1] Fan Y, Zhang Q (2013). Development of liposomal formulations: From concept to clinical
investigations. Asian J Pharm Sci.

[B2] Bozzuto G, Molinari A (2015). Liposomes as nanomedical devices. Int J Nanomedicine.

[B3] Immordino ML, Dosio L, Cattel L (2006). Stealth liposomes: review of the basic science, rationale, and
clinical applications, existing and potential. Int J Nanomedicine.

[B4] Nag OK, Awasthi A (2013). Surface engineering of liposomes for stealth
behavior. Pharmaceutics.

[B5] Nogueira E, Gomes AC, Preto A, Cavaco-Paulo A (2015). Design of liposomal formulations for cell
targeting. Colloids Surf B Biointerfaces.

[B6] Eloy JO, Petrilli R, Trevizan LNF, Chorilli M (2017). Immunoliposomes: A review on functionalization strategies and
targets for drug delivery. Colloids Surfaces B Biointerfaces.

[B7] Badiee A, Davies N, McDonald K, Radford K, Michiue H, Hart D (2007). Enhanced delivery of immunoliposomes to human dendritic cells by
targeting the multilectin receptor DEC-205. Vaccine.

[B8] Khan DR, Webb MN, Cadotte TH, Gavette MN (2015). Use of Targeted Liposome-based Chemotherapeutics to Treat Breast
Cancer. Breast Cancer (Auckl).

[B9] Li Z, Zhang L, Sun W, Ding Q, Hou Y, Xu Y (2011). Archaeosomes with encapsulated antigens for oral vaccine
delivery. Vaccine.

[B10] Fotoran WL, Santangelo RM, Medeiros MM, Colhone MC, Ciancaglini P, Barboza R (2015). Liposomes loaded with P. falciparum merozoite-derived proteins
are highly immunogenic and produce invasion-inhibiting and anti-toxin
antibodies. J Control Release.

[B11] Fotoran WL, Wunderlich G (2018). A rational design to maximize genome editing using directed
nanostructures. Curr Trends Biomed Eng Biosci.

[B12] Kumar S, Aaron J, Sokolov K (2008). Directional conjugation of antibodies to nanoparticles for
synthesis of multiplexed optical contrast agents with both delivery and
targeting moieties. Nat Protoc.

[B13] Hengen PN (1995). Purification of His-Tag fusion proteins from Escherichia
coli. Trends Biochem Sci.

[B14] Schmidt TGM, Skerra A (2007). The Strep-tag system for one-step purification and high-affinity
detection or capturing of proteins. Nat Protoc.

[B15] Fotoran WL, Colhone MC, Ciancaglini P, Stabeli RG, Wunderlich G (2016). Merozoite-protein loaded liposomes protect against challenge in
two murine models of plasmodium infection. ACS Biomater Sci Eng.

[B16] Medof ME, Nagarajan S, Tykocinski ML (1996). Cell-surface engineering with GPI-anchored
proteins. FASEB J.

[B17] Colhone MC, Silva-Jardim I, Stabeli RG, Ciancaglini P (2015). Nanobiotechnologic approach to a promising vaccine prototype for
immunisation against leishmaniasis: a fast and effective method to
incorporate GPI-anchored proteins of Leishmania amazonensis into
liposomes. J Microencapsul.

[B18] Schofield L, Hewitt MC, Evans K, Siomos MH, Seeberger PH (2002). Synthetic GPI as a candidate anti-toxic vaccine in a model of
malaria. Nature.

[B19] Tam HH, Melo MB, Kang M, Pelet JM, Ruda VM, Foley MH (2016). Sustained antigen availability during germinal center initiation
enhances antibody responses to vaccination. Proc Natl Acad Sci.

[B20] Romero CD, Varma TK, Hobbs JB, Reyes A, Driver B, Sherwood ER (2011). The toll-like receptor 4 agonist monophosphoryl lipid a augments
innate host resistance to systemic bacterial infection. Infect Immun.

[B21] Matsuo H, Yoshimoto N, Iijima M, Niimi T, Jung J, Jeong SY (2012). Engineered hepatitis B virus surface antigen L protein particles
for in vivo active targeting of splenic dendritic cells. Int J Nanomedicine.

[B22] Trager W, Jensen JB (1976). Human malaria parasites in continuous culture. Science.

[B23] Lelievre J, Berry A, Benoit-Vical F (2005). An alternative method for Plasmodium culture
synchronization. Exp Parasitol.

[B24] Lambros C, Vanderberg JP (1979). Synchronization of Plasmodium falciparum erythrocytic stages in
culture. J Parasitol.

[B25] Grimberg BT (2011). Methodology and application of flow cytometry for investigation
of human malaria parasites. J Immunol Methods.

[B26] Douglas AD, Williams AR, Illingworth JJ, Kamuyu G, Biswas S, Goodman AL (2011). The blood-stage malaria antigen PfRH5 is susceptible to
vaccine-inducible cross-strain neutralizing antibody. Nat Commun.

[B27] Santos LER, Colhone MC, Daghastanli KRP, Stabeli RG, Silva-Jardim I, Ciancaglini P (2009). Lipid microspheres loaded with antigenic membrane proteins of the
Leishmania amazonensis as a potential biotechnology
application. J Colloid Interface Sci.

[B28] Moon JJ, Suh H, Li AV, Ockenhouse CF, Yadava A, Irvine DJ (2012). Enhancing humoral responses to a malaria antigen with
nanoparticle vaccines that expand Tfh cells and promote germinal center
induction. Proc Natl Acad Sci U S A.

[B29] Medeiros MM, Fotoran WL, Dalla Martha RC, Katsuragawa TH, Pereira da Silva LH, Wunderlich G (2013). Natural antibody response to Plasmodium falciparum merozoite
antigens MSP5, MSP9 and EBA175 is associated to clinical protection in the
Brazilian Amazon. BMC Infect Dis.

[B30] Hitoshi N, Ken-ichi Y, Jun-ichi M (1991). Efficient selection for high-expression transfectants with a
novel eukaryotic vector. Gene.

[B31] Rosano GL, Ceccarelli EA (2014). Recombinant protein expression in Escherichia coli: advances and
challenges. Front Microbiol.

[B32] Çelik E, Çalik P (2012). Production of recombinant proteins by yeast cells. Biotechnol Adv.

[B33] de Bernardez Clark E (1998). Refolding of recombinant proteins. Curr Opin Biotechnol.

[B34] Kimple ME, Brill AL, Pasker RL (2004). Overview of affinity tags for protein
purification. Curr Protoc Protein Sci.

[B35] Wingfield PT (2007). Production of Recombinant Proteins. Curr Protoc Protein Sci.

[B36] Xing H, Hwang K, Lu Y (2016). Recent developments of liposomes as nanocarriers for theranostic
applications. Theranostics.

[B37] Tandrup Schmidt S, Foged C, Korsholm KS, Rades T, Christensen D (2016). Liposome-based adjuvants for subunit vaccines: formulation
strategies for subunit antigens and immunostimulators. Pharmaceutics.

[B38] Irvine DJ, Swartz MA, Szeto GL (2013). Engineering synthetic vaccines using cues from natural
immunity. Nat Mater.

[B39] Irvine DJ, Hanson MC, Rakhra K, Tokatlian T (2015). Synthetic nanoparticles for vaccines and
immunotherapy. Chem Rev.

[B40] Leserman L (2004). Liposomes as protein carriers in immunology. J Liposome Res.

[B41] Semple SC, Chonn A, Cullis PR (1996). Influence of cholesterol on the association of plasma proteins
with liposomes. Biochemistry.

[B42] Tate CG, Haase J, Baker C, Boorsma M, Magnani F, Vallis Y (2003). Comparison of seven different heterologous protein expression
systems for the production of the serotonin transporter. Biochim Biophys Acta.

[B43] Wright PE, Dyson HJ (1999). Intrinsically unstructured proteins: Re-assessing the protein
structure-function paradigm. J Mol Biol.

[B44] Morillas M, Swietnicki W, Gambetti P, Surewicz WK (1999). Membrane environment alters the conformational structure of the
recombinant human prion protein. J Biol Chem.

[B45] Weiss C, Oppliger W, Vergères G, Demel R, Jenö P, Horst M (1999). Domain structure and lipid interaction of recombinant yeast
Tim44. Proc Natl Acad Sci U S A.

[B46] Paladino S, Lebreton S, Tivodar S, Campana V, Tempre R, Zurzolo C (2008). Different GPI-attachment signals affect the oligomerisation of
GPI-anchored proteins and their apical sorting. J Cell Sci.

[B47] Kennard ML, Lizee GA, Jefferies WA, Jenkins N (1999). GPI-Anchored Fusion Proteins. Animal Cell Biotechnology. Methods in Biotechnology.

[B48] Wang J Tang S, Wan Z Gao, Y Cao Y Yi J (2016). Utilization of a photoactivatable antigen system to examine
B-cell probing termination and the B-cell receptor sorting mechanisms during
B-cell activation. Proc Natl Acad Sci U S A.

[B49] Reddy KS, Amlabu E, Pandey AK, Mitra P, Chauhan VS, Gaur D (2015). Multiprotein complex between the GPI-anchored CyRPA with PfRH5
and PfRipr is crucial for Plasmodium falciparum erythrocyte
invasion. Proc Natl Acad Sci.

[B50] Migliaccio V, Santos FR, Ciancaglini P, Ramalho-Pinto FJ (2008). Use of proteoliposome as a vaccine against Trypanosoma cruzi in
mice. Chem Phys Lipids.

[B51] Gilson PR, Nebl T, Vukcevic D, Moritz RL, Sargeant T, Speed TP (2006). Identification and stoichiometry of
glycosylphosphatidylinositol-anchored membrane proteins of the human malaria
parasite Plasmodium falciparum. Mol Cell Proteomics.

[B52] Douglas AD, Baldeviano GC, Lucas CM, Lugo-Roman LA, Crosnier C, Bartholdson SJ (2015). A PfRH5-based vaccine is efficacious against heterologous strain
blood-stage Plasmodium falciparum infection in aotus monkeys. Cell Host Microbe.

[B53] Bustamante LY, Bartholdson SJ, Crosnier C, Campos MG, Wanaguru M, Nguon C (2013). A full-length recombinant Plasmodium falciparum PfRH5 protein
induces inhibitory antibodies that are effective across common PfRH5 genetic
variants. Vaccine.

[B54] Velu V, Shetty DD, Larsson M, Shankar EM (2015). Role of PD-1 co-inhibitory pathway in HIV infection and potential
therapeutic options. Retrovirology.

[B55] Pauken KE, Wherry EJ (2015). Overcoming T cell exhaustion in infection and
cancer. Trends Immunol.

[B56] Wykes MN, Horne-Debets JM, Leow CY, Karunarathne DS (2014). Malaria drives T cells to exhaustion. Front Microbiol.

