# Supplementary material for: Production of glycosylphosphatidylinositol-anchored proteins for vaccines and directed binding of immunoliposomes to specific cell types
Source: J Venom Anim Toxins Incl Trop Dis. 2020 Aug 3;26:e20200032. doi: 10.1590/1678-9199-JVATITD-2020-0032 (PMC7401668; doi:10.1590/1678-9199-JVATITD-2020-0032)
Supplement: Additional file 1. [file 1678-9199-jvatitd-26-e20200032-s1.pdf]

J Venom Anim Toxins incl Trop Dis,
https://doi.org/10.1590/1678-9199-JVATITD-2020-0032

Supplementary Material to “Production of glycosylphosphatidylinositol-anchored proteins for vaccines and directed binding of immunoliposomes to specific cell types”

Additional file 1. Sequences of the synthesized ZZ-domain and of oligonucleotides used for murine CD14 amplification (introduced restriction sites are underlined).

Duplicated ZZ domain/Strep Tag:

ggatccTGGTCCCACCCTCAGTTCGAGAAGgtggacaacaagttcaataaggagcagcagaacgctttctacgagatcctgcacctgcccaacctgaacgaggagcaacggaacgccttcatccagagcctgaaggatgacccaagccagagcgccaacctgctggctgaggccaagaagctgaacgacgcccaggcccctaagggcggggtcgataataaatttaacaaggagcagcagaacgccttctacgagatcctgcatctccctaatctgaatgaagagcagcgcaacgccttcatccagagcctcaaggacgatcccagccagagcgctaatctgctcgccgaagctaagaaactcaatgatgctcaggctcccaaggaattc

mCD14-GPI domain primers

GPI Forward

gaattcgactggcccagtcagctaac

GPI reverse

gcggccgcttaaacaaagaggcgatctcctaggag
